# A Study of the Pattern of Admissions to the Accident and Emergency (A&E) Department of a Tertiary Care Hospital in Sri Lanka

**DOI:** 10.1155/2020/6327293

**Published:** 2020-08-13

**Authors:** Priyamali Jayasekera, Gayani Dassanayake, Kasthuri Bandara, Lakmali Jayawardhena, K. M. S. Malkanthi

**Affiliations:** ^1^General Sir John Kotelawala Defence University, Colombo, Sri Lanka; ^2^Accident and Emergency Department, Provincial General Hospital, Kurunegala, Sri Lanka

## Abstract

**Introduction:**

The latest national healthcare reform policies of Sri Lanka include the development of accident and emergency (A&E) departments in all major hospitals. Provincial General Hospital Kurunegala (PGHK) is a home to the first established A&E department in Sri Lanka. PGHK provides services to a population of 2.4 million spread out in the North Western Province and part of the Sabaragamuwa Province. This study was carried out to identify the pattern of all admissions to the A&E department of PGHK.

**Methods:**

The prospective observational study was carried out from July 1, 2016, to June 30, 2017 (one year) to identify the pattern of admissions to the A&E department.

**Results:**

There were 49,213 admissions to PGHK's A&E department during the study period. The average number of admissions was 135 (±17.9) per day. The percentage of deaths in the A&E department was 0.21%. The mean age of admitted patients was 46.7 (±21.7) years. A further 62% of admitted patients were males. The number of medical, surgical, paediatric, and gynaecological and obstetrical admissions was 55%, 42%, 3.5%, and 0.22%, respectively. Among the common emergency medical presentations, 34% were chest pain, 11% patients presented with unilateral weakness and/or slurring of speech, 10% covered dyspnoea, and 9% complained of dizziness/giddiness. Among emergency surgical presentations, 83% were trauma due to accidents, of which 27% were road-traffic-related accidents (RTAs). *Discussion*. The A&E department of PGHK provides services to a significantly high number of health emergencies every day. The majority of these admissions was due to chest pain and trauma related to accidents. The lower recorded number of paediatric and gynaecological and obstetrical emergencies presented to the A&E department is a result of a government policy mandating the admission of these types of patients directly to their respective wards. Further infrastructure development, staff recruitment, and training have to be planned and implemented to address the significantly high number of admissions to the A&E Department of PGHK.

## 1. Introduction

Sri Lanka is a South Asian island nation in the Indian Ocean and has a population of nearly 20 million (20.95 million) [1, 2]. In Sri Lanka, both public and private sectors provide health care. There are 1,104 curative care hospitals in the government health services [2]. The network of these curative institutions is made up of national hospitals, teaching hospitals, provincial general hospitals, district general hospitals, base hospitals, and peripheral units such as divisional hospitals and rural hospitals.

**Figure 1:**
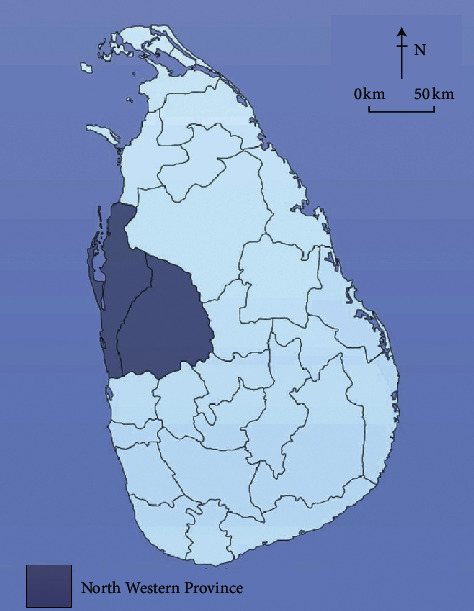
Sri Lanka.

National hospitals and teaching hospitals provide health care in the four main specialities (medicine, surgery, paediatrics, and gynaecology and obstetrics), and most provide health care in the important subspecialities (cardiology, pulmonology, ophthalmology, orthopaedic surgery, etc.). A provincial general hospital is a type of general hospital with more facilities. Each district has one general hospital (called a district general hospital) which provides care in the four main specialities and some of the subspecialities. Base hospitals provide health care only in the four main specialities. Divisional hospitals, peripheral units, and rural hospitals have a very limited number of facilities and do not provide any specialized care. The North Western Province (Figure 1), where PGHK is located, has one district general hospital, six base hospitals, and 12 divisional hospitals [3].

Accident and emergency care are demanding and complex areas of practice, presenting diverse challenges for patient-centred care. Sri Lanka has achieved impressive health status indicators which are almost comparable with those in the well-resourced world. However, accident and emergency care services need further development. The government-sanctioned national A&E policy envisages that the country will soon become a nation with a high quality of life for all of its citizens and achieve standards of living comparable to the well-resourced world. This policy mandates the development of A&E departments in all major hospitals [4].

Provincial General Hospital Kurunegala (PGHK), which was established in the late 1890s, renders services to 2.4 million people in the North Western Province and part of the Sabaragamuwa Province. It has inpatient care facilities, outpatient departments, and clinical facilities. The total bed strength is over 700. The daily Outpatient Department (OPD) attendance is over 1,500 (patients), and inpatient admissions are above 450. This hospital offers care in all the major medical specialities and subspecialities. PGHK pioneered the establishment of an Accident and Emergency department in Sri Lanka in 2002 [4]. This 14-bed unit, with staff of 42 nurses and 39 doctors, is headed by an acute care specialist and provides 24-hour services daily with full resuscitation facilities for a wide range of conditions. In-house general surgeons, orthopaedic surgeons, and other specialists provide necessary services for 24 hours every day. This study was carried out to identify the pattern of admissions to the A&E department of PGHK and to strengthen the A&E services in the country.

A&E departments collectively perform three main roles in the country: as an alternative to primary care services for the first point of contact care, as acute diagnostic and treatment centres for patients who need immediate treatment, and as portals for emergency admission to hospitals [5]. However, the main aim is to stabilize patients who have a life-threatening injury or illness and focus on the provision of immediate urgent medical intervention [6].

The three fundamental functions of a health system are to improve the health of the population, respond to people's expectations, and provide financial protection against the costs of ill-health [6]. Emergency medical care can contribute positively to these functions.

**Figure 2:**
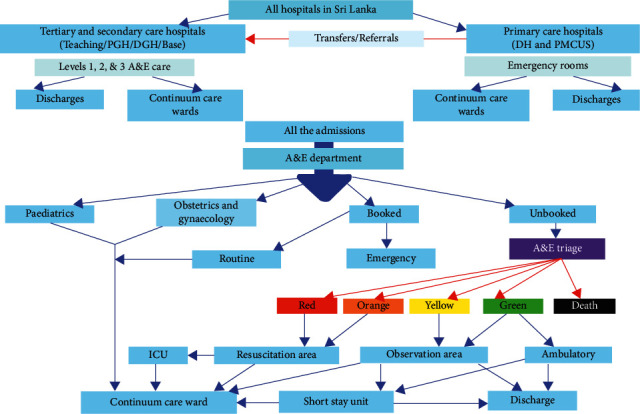
Referral system and A&E set up in Sri Lanka.

In Sri Lanka, all admissions to the hospital are processed through A&E departments (in hospitals where the A&E departments are firmly established) [4]. All patients are triaged on arrival to the A&E department. The maximum duration of stay of a patient in the A&E department is four hours after which the patient is admitted to the continuum care unit, the short stay unit (SSU), or the intensive care unit depending on care needs (Figure 2). At the time of this study, ambulance facilities were not commonly available in the country. Even now, ambulance facilities are only widely available in the Western Province of Sri Lanka.

Following an extensive search, it was concluded that there were no discoverable data on accident and emergency admissions in Sri Lanka. The amount of similar; useable data were very sparse in the rest of Asia as well. Policymakers, funders, and providers of healthcare need to have information about the epidemiology of their communities and need to understand what is happening within A&E departments to improve health services. It is paramount to study the pattern of emergencies presented to the A&E department to improve the quality of care, upgrade resources, and educate the staff in A&E settings all around the country.

### 1.1. Objectives

Our main aim was to identify the pattern of all the admissions to the A&E department at PGHK. Specifically, the objective was to identify the pattern of medical, surgical, paediatric, and gynaecological and obstetrical (Gyn&Obs) emergency admissions to the A&E department and identify common medical and surgical emergencies in the Kurunegala area.

## 2. Methods

### 2.1. Study Setting

The study was conducted in Provincial General Hospital Kurunegala (PGHK), which is situated in the North Western Province of Sri Lanka.

#### 2.1.1. Method

The prospective observational study was carried out from July 1, 2016, to June 30, 2017 (for the duration of one year) to identify the pattern of admissions to the A&E department, PGHK.

#### 2.1.2. Sample Size

The observed sample size comprised all the patients admitted to the A&E department during the above period. No specific sample size calculation was performed as the objective was to recognize the pattern of admissions.

#### 2.1.3. Inclusion Criteria

All scheduled admissions that later became emergencies and all unscheduled admissions were included in this study (Figure 2).

#### 2.1.4. Exclusion Criteria

Those who were booked for routine admissions, obstetrical and gynaecological admissions, and paediatric admissions were not included in the study. Those that were documented dead on arrival which were not attended by the A&E team for resuscitation were also excluded from the study.

#### 2.1.5. Research Tools and Data Collection Technique

The audit tool employed was the data sheet based on the International Classification of Primary Care (ICPC) and the International Classification of Diseases (ICD10). The ICPC is the most common international classification system for documenting information on primary care [7]. The ICD is considered to adequately accommodate for the needs of multiple use cases and users in the recording, reporting, and analysis of health information.

Patients usually wait in the A&E department for a maximum period of four hours during which they present various complaints. It is difficult to provide complete diagnoses within this four-hour period before transferring the patients in question to a ward or discharging them. For example, while some cases can be provided with a direct diagnosis such as ‘ST-elevation myocardial infarction,' there are other cases (vomiting and chills) where symptoms are noticeable but cannot be provided with a direct diagnosis on discharge. Therefore, both classifications (ICPC and ICD) were applied. The most recent versions were ICPC-2 which was revised in 2015 and ICD 10-2016 which was the latest version available at the time the information was recorded.

The development of the data collection forms was based on the patient flow through the A&E department and the pattern of admissions. Patients aged less than 12 years are considered the paediatric group. The first part of the data sheet consisted of demographic data, which contained the serial number, date, the admission number, age, gender, type of referral, the category of speciality (medicine, surgery, paediatric, or gynaecology and obstetrics), and the presenting complaint. The demographic data were common to all four forms except for the surgical form, where it was necessary to indicate causes such as road traffic accident, fall, assault, hanging, and drowning. In road traffic accidents, the type of vehicle or pedestrian had to be documented as well. Data collection was done manually using a special paper-based data form. There were two sections on each side of the paper: section 1 for demographic data and section 2 for diagnosis in each particular speciality.

All the nurses were trained to fill the first part of the data sheet by the principal investigator and the coinvestigator (nurse in charge). The first part was filled by the admitting nursing officer on arrival of the patient to the A&E department, and the data form was attached to the A&E admission note.

Three coinvestigators who were medical officers worked in the A&E department, and four research assistants who were preinterns were trained to gather data from the patient's clinical notes before discharging them from the A&E department while maintaining confidentiality of the patients. In addition, all medical officers (MO) in the A&E department were also trained to complete the second part of the data sheet by the principal investigator. Incomplete data sheets were completed by the coinvestigators and research assistants on the following day by gathering the data from clinical notes.

#### 2.1.6. Data Quality Control

A pilot study was carried out for the duration of one week. This was conducted to identify the deficiencies of the study—mainly to improve the data sheets and to identify logistical issues associated with the collection of data, timely entering of data into electronic databases, the secure storage of data sheets, and the staff requirements for the study. When the pilot study ended, the data sheets were amended according to the pattern of relevant admissions and face validated by experts in each field. A software database was prepared with Microsoft Access to document data via the computer.

The completion of the first part of the data sheet was supervised by the nurse in charge (coinvestigator) throughout the study, and its accuracy was confirmed by the research assistants. The accuracy of every diagnosis was also confirmed by the principal investigator who was a specialist in internal medicine. She was supported by the three coinvestigators (medical officers).

Ethical approval was obtained from the Ethical Review Committee of Provincial General Hospital Kurunegala.

### 2.2. Data Processing and Analysis

This paper-based data sheet was filled in daily. Information compiled from the collected paper-based data sheets was entered into the computer on the next day (also performed daily). The collected data were analyzed using SPSS (version 21). Descriptive statistics were generated for the pattern of admissions, presentation of complaints, and the final diagnosis at the A&E department.

## 3. Results

### 3.1. Demographic and Baseline Information

There were 49,213 admissions to the A&E department during the study period. There were 32,911 (66.9%) self-referrals and 16,034 (32.6%) transfers from hospitals. There were 255 (0.5%) referrals by specialists and general practitioners. There were 3,773 (23.5%) admissions that were transferred from base hospitals and 12,261 (76.5%) admissions transferred from other peripheral hospitals.

The average number of admissions was 135 (±17.9) per day. The number of deaths in the A&E department was 105 (0.21%). Out of these deaths, 17 were pronounced dead on arrival, while the rest expired in the A&E department. Among them, there were 100 cardiac arrests of which 24 (24%) were successfully resuscitated and transferred out of the A&E department. The mean age of admitted patients was 46.7 (±21.7) years with 62% of them being males and 38% being females, resulting in a male to female ratio of 1.6 : 1. The number of medical, surgical, paediatric, and gynaecological and obstetrical admissions was 26,825 (55%), 20,524 (42%), 1,751 (3.5%), and 113 (0.22%), respectively (Table 1).

### 3.2. Presentations to A&E

Out of the common emergency medical presentations, 9,006 (34%) were of chest pain, 2,967 (11%) patients presented with unilateral weakness and/or slurring of speech, 2,805 (10%) were of dyspnoea, 2,296 (9%) complained of dizziness/giddiness, and 1,758 (6.5%) with animal bites. About 29.5% of emergency medical presentations were of other complaints out of which the proportion of each unique complaint was less than 5%. Among them, 1,145 (4.2%) presented with self-poisoning, 619 (2.3%) with snake bites, 415 (1.5%) with features of hyperglycemia and hypoglycemia, and 351 (1.3%) with seizures. There were about 2,440 (9%) presentations which comprised simple headaches, backaches, nonspecific symptoms, and day-one fevers.

### 3.3. Pattern of Admission throughout the Year

**Figure 3:**
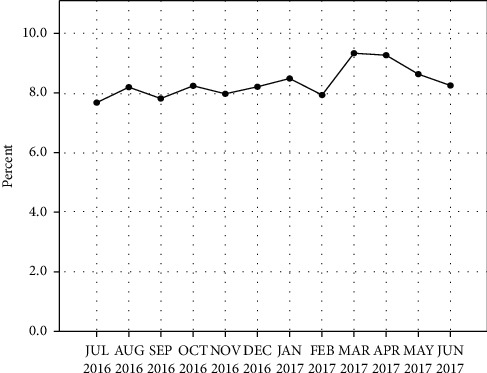
Pattern of admissions throughout the year.

The patient presentations were steady for most of the year except on the months of March and April when they peaked (Figure 3). On a daily basis, more patients arrived between 8:00 am and 12:00 pm with Monday and Friday being the busiest days of the week. There were 12,833 (26%) discharges over the year; monthly discharges numbered 1,069.4 (±162.8). Two hundred and seventeen (0.44%) patients were transferred to the country's two national hospitals located in Colombo and Kandy. Patients who were triaged to the resuscitation bay (red and orange) were seen immediately, and patients who were triaged to the observation bay (yellow and green) were seen within 30 minutes of admission. The maximum duration of stay of a patient in A&E was four hours.

### 3.4. Diagnoses at A&E: Medicine

#### 3.4.1. Cardiovascular, Ischaemic, and Circulatory Diseases

There were 9,555 (19.4%) occurrences of cardiovascular, ischaemic, and circulatory diseases presented to the A&E department, namely, acute coronary syndrome (ACS) (ST-elevation myocardial infarction (STEMI), non-ST-elevation myocardial infarction (NSTEMI), and unstable angina (UA)), angina, heart failure, cerebrovascular accidents (ischaemic stroke, haemorrhagic stroke, transient ischaemic attacks (TIA), and subarachnoid haemorrhage (SAH)), and hypertension which were among the diseases presented (Table 2).

#### 3.4.2. Subspecialities

Overall, gastrointestinal/genitourinary (GI/GU), pulmonological, infectious disease, endocrine, and neurological diagnoses were numbered at 3,221, 2,801, 902, 436, and 351, respectively. GI and GU patients were relatively younger than others. Patients that presented with gastritis were also mainly young (Table 3). In pulmonology, chronic obstructive pulmonary disease (COPD) and bronchial asthma (BA) were the most common diseases. COPD patients were also much older than bronchial asthma patients (Table 4). The number of fever patients that were categorized as infectious diseases was 902. Of these, 470 were diagnosed with viral fever, 311 with dengue fever, and 67 with sepsis. The majority of fever patients was directly sent to medical wards when they were stable on arrival. There were 436 endocrine presentations, most of which were mainly secondary to diabetic emergencies (Table 5). There were also 351 epilepsy patients presented with seizures.

#### 3.4.3. Toxicology, Snake Bites, and Animal Bites

The number of agrochemical poisoning presentations was 568 and can largely be explained by Kurunegala, being an agricultural area. However, a similar number of presentations (577) were poisoning due to self-ingestion of drugs. This can mostly be explained by considering that drug self-ingestion is the preferred mode of suicide for younger populations (Table 6). Kurunegala's agricultural makeup also accounts for the increased prevalence of snake bites in the area (Table 7). There were 1,758 dog bites that were major bites which were taken into the A&E department for initial management (Table 7).

### 3.5. Diagnoses at A&E: Surgery

**Figure 4:**
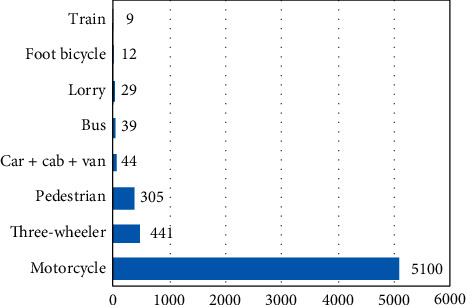
RTA-types of vehicles involved in the injury.

Among emergency surgical presentations, trauma due to accidents numbered 17,035 (83% of presentations) in which road traffic accidents (RTAs) numbered 5,444 (32% of presentations) (Table 1). The motorcycle was the most common vehicle involved in an RTA (Figure 4). The most common nontraumatic presentation was abdominal pain which accounted for 1,854 (9%) cases. The majority of abdominal pain cases presented with renal colic (777 (42%)) and acute urinary retention (257 (14%)). There were a large number of trauma cases, almost all of which were soft tissue injuries and fractures. There were more instances of upper limb injuries than lower limb injuries (6,987 vs. 6,907) (Tables 8 and 9). All the injuries had male predominance. Among upper limp injuries, there were more soft tissue injuries (4,181) than fractures (2,806). Radial fractures (991) were the most common with a male to female ratio of 2 : 1. When considering the lower limb injuries, fractures (4,769) numbered more than the soft tissue injuries (2,138), and tibial fractures (1,347) were the most common. A significant amount of facial injuries and fractures was due to assaults (Table 10). There were 353 chest injuries and 133 burns that were also presented during that period.

### 3.6. Diagnoses at A&E: Paediatrics and Gynaecology and Obstetrics

There were 1,751 (3.5%) paediatric cases. Common cases such as respiratory tract infections and gastroenteritis were directly sent to the wards, and the A&E department mainly managed animal bites involving children. Since this can lead to misinterpretation of the pattern of admissions, a separate analysis was not conducted. There were only 86 gynaecological and 27 obstetrical admissions. Common emergencies such as postpartum haemorrhages, complicated labours, and pre-eclampsia were directly admitted to wards. Among the presentations to the A&E department, the most common gynaecological emergency presented was bleeding per vagina, while the most common obstetric emergency was postpartum high blood pressure.

## 4. Discussion

The A&E department of PGHK provides services to a significantly high number of health emergencies every day. This figure closely resembles the Blackpool, United Kingdom, study [8]. The A&E admission register in PGHK shows an increase in numbers in recent years. In PGHK, all admissions to the hospital flow through the A&E department with all patients being triaged on arrival (Figure 2). The patients observed by this study were seen within 30 minutes of admission, whereas the average waiting time was 120 minutes in the study in Hong Kong [9]. The maximum duration of stay of a patient in A&E was four hours after which the patients were admitted to a continuum care unit or an intensive care unit depending on the severity of their conditions.

Presentations of patients were steady over most of the months except in March and April. This was mainly due to the traditionally festive season of the country occurring during this period which increases the occurrence of accidents. European countries found more admissions in winter months, whereas this study observed a largely steady stream of admissions except in the March-April period. The reason for the absence of a seasonal variation is the relative stability of Sri Lankan weather throughout the year. On a daily basis, more patients arrived between 8:00 am and 12:00 pm with Monday and Friday being the busiest days of the week. Generally, the number of admissions is higher on weekdays due to much of the population heading to their places of employment in between Monday and Friday. The burden is noticeably lower on Saturdays and Sundays owing to people opting to spend their weekends at home. Comparatively, the Blackpool study experienced more patients on Saturdays and less on Wednesdays.

The patients in this study, on average, were in their midforties—comparable with Indian data which describe a majority of admissions as being adults [10]. A further 62% of admitted patients were males, whereas it being around 55% in the same Indian study [10]. In the Blackpool study, however, there were no gender differences [8]. There were and have been no studies to compare the Sri Lankan figures to date. Out of the admissions, 70% were self-referrals, and 30% were transfers from hospitals. When it is compared to the Blackpool and other studies, this information is important because most patients were universally brought in by ambulances, and about one-fifth of patients were referred by general practitioners [8]. A lack of ambulance services has resulted in most of these referrals being self-administered, with most patients having to secure their own transport. It was found that medical patients in their sixties and trauma cases in their forties are common. In contrast, while a similar age group presented in the Blackpool study, actual emergencies were detected in patients aged 35–44 years and above 75 years.

The number of deaths in the A&E department was 105 (0.21%). Of these, 23% were successful resuscitations and consequently transferred out of the A&E department. This figure is 50% lower than the European figures [11].

More than half of the admissions were due to medical emergencies which is comparable to studies done in Asia [9]. The majority of these medical admissions was due to cardiac causes later diagnosed as acute coronary syndrome and comparable with some Indian data [12]. The majority of these admissions also presented with chest pain [12]. Cerebrovascular accidents also had a weight of 6% which is a significant amount, but the majority was presented out of the window period for thrombolysis. Only two patients presented in the window period were eligible. This may be explained by the unavailability of ambulances and a lack of awareness in people about thrombolysis which needs further studies for clarification. In Kerala, India (which is the most literate state in the country), 84% were unaware of symptoms and signs of stroke [13]. There was a reasonable number of major dog bites. Since minor animal bites were not taken into the A&E department for treatment, this number will only provide an idea of major bites.

Among the rest of the medical presentations, gastritis was the most common diagnosis, while respiratory diseases (COPD and BA) were the fourth most common. There were a reasonable number of fevers also presented, whereas final diagnoses mainly consisted of viral fever, dengue fever, and sepsis, but these figures cannot be compared as stable patients were directed to medical wards rather than referred to the A&E department. However, in an Indian study done in Kerala, the most common presentation to the A&E department was fever [14]. A drug overdose is also a common mode of self-poisoning in this study and is less common in India than agrochemical poisoning [14]. This can mostly be explained by considering that drug self-ingestion is the preferred mode of suicide for younger populations. In the Blackpool study, poisoning was one of the common emergencies. In Kurunegala, an agricultural area, presentations with self-ingestion of agrochemical poison and snake bites were also common. Hump-nosed viper bites and unknown bites were the most common among snake bites. However, in India, elapid (cobra) bites were the commonest [15]. About 9% of presentations comprised simple headaches, backaches, nonspecific symptoms, and day-one fevers—all of which did not necessitate emergency care. Comparatively, in the Blackpool study, 70% of nonemergencies were directed to the emergency department. This may also be explained by the universal availability of free ambulance services in European countries, as opposed to Sri Lanka, where in most cases, the population needs to secure their own transport in the event of an emergency.

Among surgical presentations, the biggest proportion consisted of trauma cases, where figures were similar to Indian study observations, which found that the most common emergencies were trauma-related but were the result of road traffic accidents (RTAs) [10]. In this study, however, non-RTAs (2/3) are more common than RTAs (1/3). A retrospective analysis performed in Sri Lanka on road traffic crashes from 1938 to 2013 has indicated that annual crashes are on the increase, but have not assessed other traumas [16, 17]. When considering RTAs, it was discovered that motorcycle crashes made up the largest proportion of accidents, and three-wheelers accounted for the second largest amount of accidents [17–19]. These results were supported by Sri Lankan studies [17–19].

Most of the trauma-related injuries comprised upper limb and lower limb injuries. There was an average amount of head and facial injuries as well. There were more fractures found on lower limbs compared to more soft tissue injuries in upper limbs. In past studies done in Sri Lanka, extremity injuries were common. However, these studies are of a much smaller scale when compared to this one-year study [19]. A fair amount of facial injuries was due to assaults, of which most of the victims were males. Those facial injuries were also mainly soft tissue injuries (soft tissue injuries : fractures-6 : 1 ratio). Head injuries also accounted for a significant number of soft tissue injuries, skull fractures, and intracranial haemorrhages, but these figures were less comparable to global figures [20]. A significant factor was that the male victims of head injuries were in their early thirties. A significant observation is that the presented neck of femur fracture cases were mainly the result of falls and comprised patients' ages of 65 and 98 years. This age is similar to international figures [21]. All the fractures were common in males, with tibial fractures being the most common—similar to a Nigerian study [22] and global figures [20]. Observation of radial fractures had a male to female ratio of 2 : 1, with it also being the most common fracture in females.

Though abdominal pain is a regular medical complaint, 9% of cases were determined to have surgical causes with renal colic being the most common. This is comparable with Indian studies [10].

The lower recorded number of paediatric, gynaecological, and obstetrical emergencies presented to the A&E department is a result of a national A&E policy to admit such patients directly to their respective wards unless there is a real emergency. In paediatric presentations, only animal bites, seizures, and acute wheeze cases were taken into the A&E department. As a result, calculations were not done as they would have provided a wrong interpretation of the pattern of admissions. In other studies, gastroenteritis and respiratory infections were common in paediatrics though attempts at direct comparison were difficult as a majority of PGHK's patients were directly sent to wards [23]. The most common presentation of gynaecological patients was bleeding per vagina, while high blood pressure presentations numbered the highest in obstetric patients. These figures do not interpret the true pattern of admissions as they were also sent to wards/theaters directly.

A good number of admissions are unavoidable, and the A&E department is unable to prevent them. Fractures, chest pain, infections, and other emergencies are common occurrences which result in unexpected presentations to the A&E department. However, a well-managed patient with chronic diseases such as diabetes, ischaemic heart disease, or asthma should not be attending an A&E department if their primary care setting is well developed. A lack of well-developed primary health care settings is responsible for most unnecessary admissions.

Clinically stable patients with acute problems who require hospital care for conditions such as community-acquired pneumonia would see an outpatient department and be directly admitted to a medical ward rather than risking unnecessary crowding of the A&E department. Alternatively, the trend could be driven by changes in the organization of medical services in the A&E department that favour rapid diagnostic technologies and early treatment. The diagnostic services have improved, and an expectation of rapid, accurate diagnosis and treatment has become standardized, such as primary intervention for ST-segment-elevation myocardial infarction and stroke thrombolysis after imaging. The evaluation of common symptoms such as chest pain, abdominal pain, and dyspnoea has become de facto reasons for A&E referrals [24].

## 5. Recommendations

Acute coronary syndrome (ACS) is a common and important medical emergency presentation in A&E departments. Optimum infrastructure facilities and adequate training of health staff are important to prompt evaluation and management of these patients. A dedicated chest pain unit will help to reduce the burden of ACS in the A&E department. Rapid diagnostic facilities should be available in emergency care such as point-of-care troponin, arterial blood gases, full blood count, and imaging. Guidelines and algorithms for common presenting problems should be developed, tested, and agreed upon for use in A&E departments by all grades of staff and particularly aimed at those in training.

Emergency departments handle a significant number of strokes every day. There is a need for early detection with public awareness to achieve an early diagnosis of a stroke with the aim of improving the outcome. Increasing the effectiveness and availability of ambulance services is also an aspect which can be improved upon for stroke services, where patients can be directly transported without having to secure their own transport. Further studies are needed to assess the knowledge about stroke.

Trauma makes up a significant amount of surgical presentations. When it comes to trauma, instead of simply presenting and transferring patients to the relevant wards after meeting their immediate care needs, there should be a mechanism to fix single, uncomplicated fractures at the A&E department before discharging within a few hours of their arrival. Further infrastructure development, a dedicated in-house trauma team, staff recruitment, and training have to be planned. With nonspecialists providing much of the orthopaedic and other trauma and surgical care, continuing education courses becomes an important opportunity to strengthen such care [20].

Nonurgent patients could be treated in other health care settings such as primary care units in order to reduce the overburden of the A&E department. There is a need to establish a well-developed primary health care system.

Following careful observation and analysis of high-capacity operating times in the A&E department, potentially busy days and months should be the target of staff increases and other facilitative increases.

## Figures and Tables

**Table 1:** Frequency of presentations to A&E.

Speciality	Total	Males	Females
1. Medicine	26,825 (55%)	14,508 (54.0%)	12,317 (46.0%)
2. Surgery	20,524 (42%)	14,836 (72.3%)	5,688 (27.7%)
2.1. Trauma non-RTA (falls and other)	11,768 (57%)	8,311 (70.6%)	3,457 (29.4%)
2.2. RTA	5,444 (27%)	4,308 (79.1%)	1,136 (20.9%)
2.3. Assaults	1,867 (9%)	1,376 (73.7%)	491 (26.3%)
2.4. Abdominal pain (surgery)	1,445 (7%)	841 (58.2%)	604 (41.8%)
3. Paediatrics	1,751 (3.5%)	1,050 (60.0%)	701 (40.0%)
4. Gyn&Obs	113 (0.2%)		
Total	49,213		

**Table 2:** Cardiovascular, ischaemic, and circulatory diseases.

Diagnosis	Presentation	Total	Male: age	Female: age	Deaths
1. Acute coronary syndrome	Chest pain (95.5%)	*n* = 3,436 (7%)^*∗*^	*n* = 1,884 (54.8%)	*n* = 1,552 (45.2%)	13
	Dyspnoea/dizziness/giddiness (4.5%)	9.4 ± 4.3 days	62.5 ± 12.3 years	64 ± 12.4 years	
1.1. ST-elevation myocardial infarction	Chest pain (96.7%)	*n* = 695 (20.3%)	*n* = 466	*n* = 229	11
	Dyspnoea (3.2%)		59.4 ± 12.6 years	61.9 ± 12.7 years	
1.2. Non-ST-elevation myocardial infarction	Chest pain (93.4%)	*n* = 874 (25.4%)	*n* = 427	*n* = 447	1
	Dyspnoea/dizziness/giddiness (3.6%)		64.3 ± 12.5 years	64.3 ± 12 years	
1.3. Unstable angina	Chest pain (98.2%)	*n* = 1,864 (54.3%)	*n* = 991	*n* = 876	1
			64.2 ± 12.3 years	63.1 ± 11.9 years	
2. Angina	Chest pain (98.6%)	*n* = 2,490 (5.05%)^*∗*^	*n* = 1364	*n* = 1126	0
			57.9 ± 11 years	60.2 ± 11.0 years	
3. Heart failure	Chest pain (62.5%)	*n* = 496 (1%)^*∗*^	*n* = 235	*n* = 261	9
	SOB (32.1%)		67.2 ± 13.3 years	69.3 ± 13.3 years	
	Others (5.4%)				
4. Hypertension	Hypertension (26.5%)	*n* = 166 (0.3%)	*n* = 73	*n* = 93	0
	Dizziness/giddiness (30.1%)		59 ± 14.8 years	60 ± 16 years	
	Chest pain (15.7%)				
	Others (27.7%)				
5. Cerebrovascular accidents	Unilateral weakness (74.3%)	*n* = 2967 (6%)^*∗*^	*n* = 1,705 (57.5%)	*n* = 1,262 (42.5%)	10
	Slurring of speech (10.8%)	8.8 ± 3.3/per day	63.8 ± 14.4 years	66.7 ± 14.2 years	
	Mixed (10.6%)	65 ± 14.4 years			
	Loss of consciousness (4.2%)				
5.1. Ischaemic stroke		*n* = 2,073 (70%)	*n* = 1,191	*n* = 882	5
			67.5 ± 13.2 years	64.7 ± 13.4 years	
5.2. Haemorrhagic stroke		*n* = 413 (14%)	*n* = 233	*n* = 180	4
			64 ± 17 years	67.7 ± 15.1 years	
5.3. Transient ischaemic attack		*n* = 446 (15%)	*n* = 261	*n* = 185	0
			61 ± 14.7 years	67.7 ± 15.1 years	
5.4. Subarachnoid haemorrhage		*n* = 35 (1%)	*n* = 20	*n* = 15	1
			50.5 ± 19.7 years	51 ± 19.7 years	

^∗^from overall admissions.

**Table 3:** Gastrointestinal/genitourinary disease.

Diagnosis	Total	Male: age	Female: age
All GI/GU diseases	*n* = 3,221	*n* = 1,741 (54%)	*n* = 1,480 (45.9%)
		46 ± 17.3 years	45.1 ± 19.2 years
Gastritis	*n* = 2,187 (67.9%)	*n* = 1,005 (45.9%)	*n* = 1,182 (54.1%)
Vomiting	*n* = 338 (10.9%)	*n* = 143 (42.3%)	*n* = 195 (57.7%)
Chronic kidney disease	*n* = 234 (7.3%)	*n* = 151 (64.5%)	*n* = 83 (35.5%)
Haematemesis and melena	*n* = 176 (5.5%)	*n* = 125 (71%)	*n* = 51 (28.9%)
Urinary tract infection	*n* = 110 (3.4%)	*n* = 50 (45.4%)	*n* = 60 (54.5%)
Diarrhoea	*n* = 70 (2.2%)	*n* = 25 (35.7%)	*n* = 45 (64.3%)
Acute kidney injury	*n* = 41 (1.3%)	*n* = 26 (63.4%)	*n* = 15 (36.6%)
Others	*n* = 65 (2%)		

**Table 4:** Pulmonology.

Diagnosis	Total	Male: age	Female: age
All respiratory diseases	*n* = 2,801	*n* = 1,607 (57.3%)	*n* = 1,194 (42.6%)
		60.4 ± 17.2 years	59.4 ± 19.2 years
COPD	*n* = 1,168 (41.7%)	*n* = 767 (65.6%)	*n* = 401 (34.3%)
		67 ± 11.8 years	68 ± 13.87 years
Bronchial asthma	*n* = 1,067 (38.1%)	*n* = 509 (47.7%)	*n* = 558 (52.3%)
		53 ± 19.7 years	53 ± 20.3 years
Pneumonia	*n* = 381 (13.6%)	*n* = 210 (55.1%)	*n* = 171 (44.9%)
		60 ± 15.7 years	64 ± 16.1 years
Bronchitis	*n* = 135 (4.8%)	*n* = 86 (63.7%)	*n* = 49 (36.3%)
		51 ± 17.3 years	50 ± 22 years
Epistaxis	*n* = 22 (0.8%)		
Pleural effusion	*n* = 15 (0.5%)		
Others	*n* = 13		

**Table 5:** Endocrinology.

Diagnosis	Total	Male: age	Female: age
All endocrine diseases	*n* = 436	*n* = 229 (52.5%)	*n* = 207 (47.5%)
		55.9 ± 16 years	57.2 ± 19.3 years
Hypoglycemia	*n* = 287 (65.8%)	*n* = 150 (52.3%)	*n* = 137 (47.7%)
Hyperglycemia	*n* = 128 (29.4%)	*n* = 64 (50%)	*n* = 64 (50%)
Hyperkalemia	*n* = 9		
Hypokalemia	*n* = 6		
Hyponatremia	*n* = 1		
Hypocalcaemia	*n* = 1		
Others	*n* = 4		

**Table 6:** Toxicology.

Diagnosis	Total	Male: age	Female: age
All poisoning	*n* = 1,145	*n* = 564 (49.3%)	*n* = 581 (50.7%)
		33.8 ± 16.7 years	26 ± 12.4 years
Drug	*n* = 577 (50.4%)	*n* = 200 (34.7%)	*n* = 377 (65.3%)
Plant	*n* = 331 (28.9%)	*n* = 192 (58%)	*n* = 139 (42%)
Pesticide	*n* = 131 (11.4%)	*n* = 105 (80.2%)	*n* = 26 (19.8%)
Others	*n* = 61 (5.3%)	*n* = 34 (55.7%)	*n* = 27 (44.3%)
Weedicide	*n* = 45 (3.9%)	*n* = 33 (73.3%)	*n* = 12 (26.7%)

**Table 7:** Snake bites and animal bites.

Diagnosis	Total	Male: age	Female: age
Snake bites	*n* = 623	*n* = 381 (61.2%)	*n* = 242 (38.8%)
		42.7 ± 17 years	46.6 ± 16.4 years
Hump nose viper bites	*n* = 295 (47.35%)	*n* = 169 (57.3%)	*n* = 126 (42.7%)
Unknown snake bites	*n* = 251 (40.4%)	*n* = 156 (62.1%)	*n* = 95 (37.8%)
Russell's viper bites	*n* = 60 (9.63%)	*n* = 45 (75%)	*n* = 15 (25%)
Cobra bites	*n* = 10 (1.6%)	*n* = 8 (80%)	*n* = 2 (20%)
Centipede bites	*n* = 4	*n* = 3	*n* = 1
Krait bites	*n* = 2	*n* = 1	*n* = 1
Rat snake bites	*n* = 1		
Dog bites (major)	*n* = 1,758	*n* = 914 (52%)	*n* = 844 (48%)
	4.8 ± 1.4 per day	41.8 ± 18.6 years	45 ± 18.4 years

**Table 8:** All upper limb injuries.

Diagnosis	Total	Male: age	Female: age
All upper limb injuries	*n* = 6,977	*n* = 5,198 (74.5%)	*n* = 1,779 (25.5%)
		35 ± 18.2 years	40.4 ± 21.2 years
Soft tissue injuries	*n* = 4,181 (60%)	*n* = 3,109 (74.5%)	*n* = 1,062 (25.4%)
Fractures	*n* = 2,806 (40%)	*n* = 2,089 (74.4%)	*n* = 717 (25.5%)
Clavicle fracture	*n* = 240	*n* = 185 (77%)	*n* = 55 (22.9%)
		39.2 ± 18.9 years	36.6 ± 17.1 years
Humerus fracture	*n* = 205	*n* = 138 (67.8%)	*n* = 66 (32.2%)
		28.9 ± 22.5 years	42.2 ± 19.8 years
Radius and ulnar fracture	*n* = 133	*n* = 107 (80.4%)	*n* = 26 (19.5%)
		31.3 ± 18 years	38.4 ± 25.2 years
Radius fracture	*n* = 991	*n* = 666 (67.2%)	*n* = 325 (32.8%)
		33 ± 19.6 years	45.7 ± 22.1 years
Ulnar fracture	*n* = 422	*n* = 331 (78.4%)	*n* = 91 (21.7%)
		35.8 ± 18.7 years	40.9 ± 21.5 years
Scapula fracture	*n* = 34	*n* = 27 (79.4%)	*n* = 7 (20.6%)
		33.9 ± 15.9 years	50 ± 22.1 years
Hand fracture	*n* = 781	*n* = 634 (81.2%)	*n* = 147 (18.8%)
		37.6 ± 17.4 years	40.5 ± 18.8 years
Road traffic accidents	*n* = 2,406 (34.5%)		
Other accidents	*n* = 4,571 (65.4%)		

**Table 9:** All lower limb injuries.

Diagnosis	Total	Male: age	Female: age
All lower limb injuries	*n* = 6,907	*n* = 5,185 (75%)	*n* = 1,721 (24.9%)
		37.7 ± 18 years	46.6 ± 22.4 years
Soft tissue injuries	*n* = 2,138 (31%)	*n* = 1,622 (76%)	*n* = 516 (24%)
Fractures	*n* = 4,769 (69%)	*n* = 3,563 (75%)	*n* = 1,206 (25%)
Pelvic fracture	*n* = 88	*n* = 64 (72.7%)	*n* = 24 (27.3%)
		45.4 ± 23.0 years	64.2 ± 23.2 years
Femur	*n* = 717	*n* = 427 (59.6%)	*n* = 290 (40.6%)
		47.3 ± 23.4 years	67.6 ± 18.6 years
Neck of femur fracture	*n* = 300	*n* = 108 (36%)	*n* = 192 (64%)
		65 ± 19.8 years	74 ± 12.3 years
Fibula fracture	*n* = 774	*n* = 624 (80.6%)	*n* = 150 (19.4%)
		37.2 ± 15.2 years	43.6 ± 19.9 years
Tibial fracture	*n* = 1,337	*n* = 1,085 (81.3%)	*n* = 252 (18.7%)
		37.7 ± 16.1 years	43.1 ± 18.9 years
Tibia and fibula fracture	*n* = 544	*n* = 446 (82%)	*n* = 98 (18%)
		41.1 ± 15 years	37.4 ± 18 years
Foot fracture	*n* = 726	*n* = 579 (79.8%)	*n* = 147 (20.2%)
		37.9 ± 16.9 years	42.5 ± 17.7 years
Spinal injury	*n* = 283	*n* = 230 (81.2%)	*n* = 53 (18.7%)
		51.6 ± 22 years	48.4 ± 16.9 years
Road traffic accidents	*n* = 2,406 (34.5%)		
Other accidents	*n* = 4,571 (65.4%)		

**Table 10:** Head injuries and facial injuries.

Diagnosis	Total	Male: age	Female: age
Head injury	*n* = 3,514	*n* = 2,466 (70.2%)	*n* = 1,048 (29.8%)
		32.6 ± 22 years	36.6 ± 26.1 years
Facial injury	*n* = 3,505	*n* = 2,532 (72.3%)	*n* = 973 (27.8%)
		35 ± 17.7 years	39 ± 20.3 years
Soft tissue injuries	*n* = 2,956 (84.3%)	*n* = 2,116 (71.6%)	*n* = 840 (28.4%)
Facial bone fracture	*n* = 549 (15.7%)	*n* = 416 (75.8%)	*n* = 133 (24.2%)
Road traffic accidents	*n* = 205 (5.8%)		
Other accidents	*n* = 2,055 (58.5%)		
Assaults	*n* = 590 (16.8%)		

## Data Availability

All the data are available in the electronic and hard format.

## Additional Points

Patients remain for a limited time in the A&E department, and only provisional diagnoses are provided. Diagnoses at the A&E department could change subsequently once other investigations are available. As paediatric and gynaecological and obstetrical admissions are directly admitting to wards without going through the A&E departments, it is difficult to formulate a proper pattern of admissions for these two specialities. Outcomes excepting mortality and cardiac arrests have not been measured.
